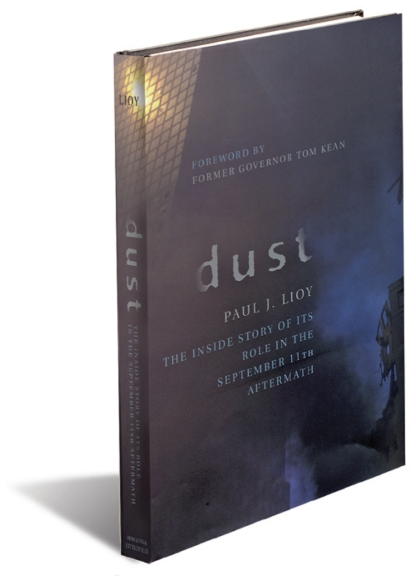# Dust: The Inside Story of Its Role in the September 11th Aftermath

**Published:** 2010-05

**Authors:** Timothy J. Buckley

**Affiliations:** *Timothy J. Buckley is an associate professor and Chair of the Division of Environmental Health Sciences at The Ohio State University (OSU) College of Public Health. He is trained in Exposure Science and Industrial Hygiene and has over 20 years of environmental health research experience. Paul J. Lioy was Buckley’s doctoral dissertation advisor (1991) and they both serve as Associate Editors for* Environmental Health Perspectives

*Dust: The Inside Story of Its Role in the September 11th Aftermath* provides a first-hand account of this country’s response to the environmental health threat following the 11 September 2001 (9/11) attack on the World Trade Center (WTC). This important story could not have been told with as great an understanding until now. The 9 ensuing years have allowed the emotional distance necessary for a thoughtful analysis, providing both perspective and the time needed for health effects from long and short-term exposures to be revealed.

In a few short hours, the WTC was reduced to billions of tons of dust—aerosolized particles composed of building materials that included heavy metals, asbestos and mineral fibers, along with by-products of combustion—that blanketed the surrounding densely populated community. Lioy describes the successes and failures of this country’s environmental health science response in recognizing, evaluating, and controlling the threat to worker and community health. In the rush to fill the informational void in the hours and days after the attack, there were missteps in risk communication and failures in ensuring respiratory protection for workers. Among the successes occurring with time was a well-coordinated response from regional academic research centers. While focusing on the science, Lioy weaves in the political and human influences involved in allocating scarce resources amid complex stakeholder interests and participation, including community, union, academic, federal, state, and local organizations.

As the Director of the Exposure Science Division at the Environmental & Occupational Health Sciences Institute at Rutgers University, Lioy seems well positioned to tell this story. One of this country’s foremost environmental health scientists and a founder of “exposure science,” he was in the thick of the response as one of the first environmental health scientists on the scene. For years afterward, he worked to understand and evaluate the health threat. In *Dust* he distills the complex science of aerosol chemistry and physics that underlies the environmental health threat into easily understood lay terms. His description of the science and politics inherent in the aftermath comes alive as he shares how the terrorist attack affected him both personally and professionally, no doubt striking a chord with many American readers.

Lioy’s discussion of the 9/11 aftermath is founded on the WTC dust and exposure science. Lioy provides a comprehensive assessment of the dust and its chemical and physical makeup, and in so doing, informs the health threat: *a*) the transport of dust in the air and the resulting community and worker exposure, *b*) the extent and location of lung deposition and the resulting dose, and *c*) the toxicity of the dust. In particular, Lioy ably describes the environmental health threat that ensued during those first hours and days following 9/11 when the public health response was ill prepared for an event of such magnitude and hindered by priorities of recovery, leaving little physical evidence other than the dust. Because this dust covered the surrounding community and permeated homes and office buildings, Lioy’s analysis also reveals the “legacy” threat stemming from the long-term presence of the WTC dust within the community, homes, and offices. His characterization of this threat played an important role in motivating and informing indoor clean-up efforts and procedures, thereby minimizing further risk to residents.

It is apt that Lioy writes for a broad audience that might comprise scientists and nonscientists, policy makers and communities, graduate students and undergraduates. Many lessons can be learned from the response to the WTC disaster, not the least of which are how to be better prepared and how to manage risk communication, as well as the value of a well-developed and effective public health infrastructure. Accordingly, the long-term benefit of Lioy’s analysis should have increased relevance for the future. An understanding of the environmental impact of the WTC disaster is critical in helping responders understand and better prepare for future environmental disasters, in hopes of lessening the public health threats that arise. When the next environmental disaster occurs, thanks to Lioy and the many others like him who have worked tirelessly to evaluate and mitigate the WTC environmental health threat, our response will be quicker and better coordinated, grounded in a better understanding of the science, and ultimately more effective in minimizing risks to workers and communities. Lioy states that he wrote the book for his grandchildren, but the impact of his work will strike a much broader readership, both today and well into the future.

## Figures and Tables

**Figure:**